# Global trends in research of fibroblasts associated with rheumatoid diseases in the 21st century: A bibliometric analysis

**DOI:** 10.3389/fimmu.2023.1098977

**Published:** 2023-02-10

**Authors:** Runzhi Huang, Minghao Jin, Yifan Liu, Yuwei Lu, Mengyi Zhang, Penghui Yan, Shuyuan Xian, Siqiao Wang, Hao Zhang, Xinkun Zhang, Shaofeng Chen, Bingnan Lu, Yiting Yang, Zongqiang Huang, Xin Liu, Shizhao Ji

**Affiliations:** ^1^ Department of Burn Surgery, the First Affiliated Hospital of Naval Medical University, Shanghai, China; ^2^ Research Unit of Key Techniques for Treatment of Burns and Combined Burns and Trauma Injury, Chinese Academy of Medical Sciences, Beijing, China; ^3^ Shanghai Jiao Tong University School of Medicine, Shanghai, China; ^4^ Department of Orthopedics, The First Affiliated Hospital of Zhengzhou University, Zhengzhou, China; ^5^ Tongji University School of Medicine, Shanghai, China; ^6^ Department of Orthopedics, Naval Medical Center of People's Liberation Army (PLA), Second Military Medical University, Shanghai, China; ^7^ Department of Orthopedics, the First Affiliated Hospital of Naval Medical University, Shanghai, China; ^8^ Department of Rheumatology and Immunology, Second Affiliated Hospital of Naval Medical University, Shanghai, China

**Keywords:** rheumatoid diseases, fibroblasts, systemic sclerosis, rheumatoid arthritis, fibroblast-like synoviocytes, bibliometrics

## Abstract

**Background:**

Rheumatoid Diseases (RDs) are a group of systemic auto-immune diseases that are characterized by chronic synovitis, and fibroblast-like synoviocytes (FLSs) play an important role in the occurrence and progression of synovitis. Our study is the first to adopt bibliometric analysis to identify the global scientific production and visualize its current distribution in the 21st century, providing insights for future research through the analysis of themes and keywords.

**Methods:**

We obtained scientific publications from the core collection of the Web of Science (WoS) database, and the bibliometric analysis and visualization were conducted by Biblioshiny software based on R-bibliometrix.

**Results:**

From 2000 to 2022, a total of 3,391 publications were reviewed. China is the most prolific country (n = 2601), and the USA is the most cited country (cited 7225 times). The Center of Experimental Rheumatology at University Hospital Zürich supported the maximum number of articles (n = 40). Steffen Gay published 85 records with 6263 total citations, perhaps making him the most impactful researcher. Arthritis and Rheumatism, Annals of Rheumatic Diseases, and Rheumatology are the top three journals.

**Conclusion:**

The current study revealed that rheumatoid disease (RD)-related fibroblast studies are growing. Based on the bibliometric analysis, we summarized three important topics: activation of different subsets of fibroblasts; regulation of fibroblast function; and *in vitro* validation of existing discoveries. They are all valuable directions, which provide reference and guidance for researchers and clinicians engaged in the research of RDs and fibroblasts.

## 1 Introduction

Rheumatoid Diseases (RDs) refer to a large group of diseases that encroach on joints and surrounding tissues, most of which are auto-immune diseases and have a genetic tendency. The onset of the disease is often covert and slow, often involving intermittent or migratory pain, stiffness, and swelling of joints and muscles. The course of diseases is long-lasting. As the disease progresses, systemic symptoms such as myalgia, fatigue, low fever, weight loss, and depression may develop. If not treated sufficiently in time, it can lead to joint malalignment, bone erosion, cartilage destruction, and ultimately disability. Also, systemic symptoms such as atherosclerosis and vasculitis can be fatal (1).

According to the ICD-10 (International Classification of Diseases-10), RDs cover more than 100 diseases, but clinically, they can be divided into four categories: CTD (connective tissue disease), SpA (spondylarthritis), osteoarthritis, and crystalline arthritis. The incidence rate, onset age, and sex tendency vary in different types of diseases. CTD is the most common RD, including rheumatoid arthritis (RA) (affecting 400 - 1300 per 100,000 people worldwide (2)), lupus erythematosus (affecting 20-150 per 100,000 people worldwide (3)), systemic sclerosis (SSc) (affecting 40-340 per 100,000 people worldwide (4)), Sjögren’s syndrome (affecting 10-40 per 100,000 people worldwide (5)), and so on. Therefore, RDs have become a huge social and economic burden.

Fibroblasts, mostly characterized by the expression of markers including collagen I α chain (COL1A), PDGFRα, and THY-1 (6), are indispensable to the fibrosis process (7). Researchers have known since 1985 that fibroblasts appear within 24 hours of wound formation. They can attach to the fibrin matrix of a blood clot, proliferate, and produce collagen, glycoproteins, and mucopolysaccharides that make up the matrix (8). In addition, some fibroblasts, called myofibroblasts, produce contractile proteins, which have the ability to pull the edges of wounds together. These functions of fibroblasts make them essential for granulation tissue growth (9). In normal physiological conditions, myofibroblasts are eliminated by apoptosis as the repair scars form. However, in some pathologic situations, myofibroblasts persist and lead to excess fibrosis and wound contraction, which can interfere with normal organ function or lead to skin keloids (10). With the development of single cell analysis, heterogeneity in fibroblasts has been proven and widely accepted according to different development stages, tissue environment, and tissue origins (11, 12). Various subsets of fibroblasts are involved in many diseases due to their aberrant secretory function and apoptosis.

The pathogenesis of RDs is often complex, and the etiology is still unclear, involving a variety of genetic, environmental, and immune factors. RDs share an autoimmune-related adaptive response, while abnormal fibroblasts mainly play a major role in the progression of RA and SSc. In SSc, genetic factors and some possible infections (especially viral infections) may undermine the immune response. Multiple cytokines and growth factors, including TGF-β, IL-6, and IL-1, are released after autoantibodies injure the blood vessel endothelium, over-activating fibroblasts and leading to SSc fibrosis (13). In RA, the initiating event may be that environmental factors stimulate mucosal surfaces or lymphoid organs, inducing the conversion of arginine in mucosal proteins to citrulline. The modulated proteins are then processed by antigen-presenting cells and presented to T cells. Through IL-10 and B lymphocyte stimulator (BLyS), B cells are activated to form anti-citrulline antibodies and a range of inflammatory factors (14). Until now, these changes may not have led to clinical symptoms. Autoantibodies and inflammatory factors cause immune complexes to deposit in the synovial membrane of the joint or increase synovial vascular permeability, so as to activate synovial fibroblasts, converting them into fibroblast-like synoviocytes (FLSs, also referred to as rheumatoid arthritis synovial fibroblasts or type B synoviocytes) (15, 16). Furthermore, scientists have found that FLS can be stimulated by GSDMD and LDH expressed by monocytes to release intracellular proinflammatory factors through pyroptosis, leading to a stronger inflammatory response (17). Thereby, FLSs promote the occurrence and long-term existence of arthritis through a series of cytokines, matrix metalloproteinases, etc. In summary, aberrant fibroblasts in RDs may not be directly related to etiology, but their ability to escape from apoptosis, recruit inflammatory cells, promote pathological fibrosis, synthesize destructive proteins, activate osteoclasts, and migrate throughout the body contributes greatly to the final clinical features (18).

Most current treatments for RDs rely on immunosuppressants, non-steroidal anti-inflammatory drugs (NSAIDs), and glucocorticoids (19, 20). Taking RA as an example, active RA requires glucocorticoids to suppress inflammation and NSAIDs to relieve pain, while the treatment of the disease itself requires the early use of disease-modifying antirheumatic drugs (DMARD). Traditional DMARDs include methotrexate, hydroxychloroquine, leflunomide, and sulfasalazine. With the advancement of drug research, we can target cytokines or receptors, such as infliximab inhibiting TNF-α, tocilizumab antagonizing IL-6 receptors, rituximab depleting B cells, and tofacitinib inhibiting JAK-STAT signaling (21). However, existing treatments still focus on inhibiting the erosion and inflammation caused by autoantibodies, and few directly intervene in the process of fibrosis caused by pathological fibroblasts. The impairment of immunity to infection often results in a poor prognosis. Although glucocorticoids may be able to slow down fibrosis (22, 23), their mechanism is still unclear, and their side effects, such as decreased immunity, coagulation dysfunction, etc., can greatly affect the normal life of patients. Therapies that target fibroblasts may be able to bypass immune suppression. It may also be used as an alternative strategy for patients who are resistant to traditional treatments, or as a supplement to achieve better efficacy. Given the huge potential, studies on fibroblasts in RDs have been growing rapidly in the 21st century. Therefore, we performed this bibliometric analysis in RDs and fibroblasts.

Bibliometric research refers to an interdisciplinary science that uses mathematical and statistical methods to quantitatively analyze all the literature over a certain field in a certain period of time so as to have an intuitive understanding of the history, present, and future of the field (24). Bibliometrics can process more articles at a time than traditional review articles and predict future directions more accurately due to its unique clustering algorithm (25). Because of the need for complete reference data to analyze the influence of the articles, we chose the Web of Science database as the source of literature retrieval (26). Web of Science, being the most comprehensive database of academic resources, collects more than 21,000 high-impact journals and more than 30,000 conferences all over the world. It covers almost all the fields in biomedicine, arts, engineering, humanity, and natural science, which makes our assessment of influence more considerate and accurate (27). So far, there is no bibliometric study on fibroblasts and rheumatic diseases, but fibroblast-induced fibrosis is a very important part of RD, and there are many studies of mixed quality in this field that confuse new researchers. We conducted bibliometric analysis in the study of 3391 articles on fibroblasts and RDs to summarize the current research findings and the most influential countries, authors, institutions, and journals. In this bibliometric study, we hope that our visual processing and analysis can inform researchers about the hotspots and future trends in this field, which can inspire more high-quality research.

## 2 Material and methods

### 2.1 Search strategy

**Figure 1:**
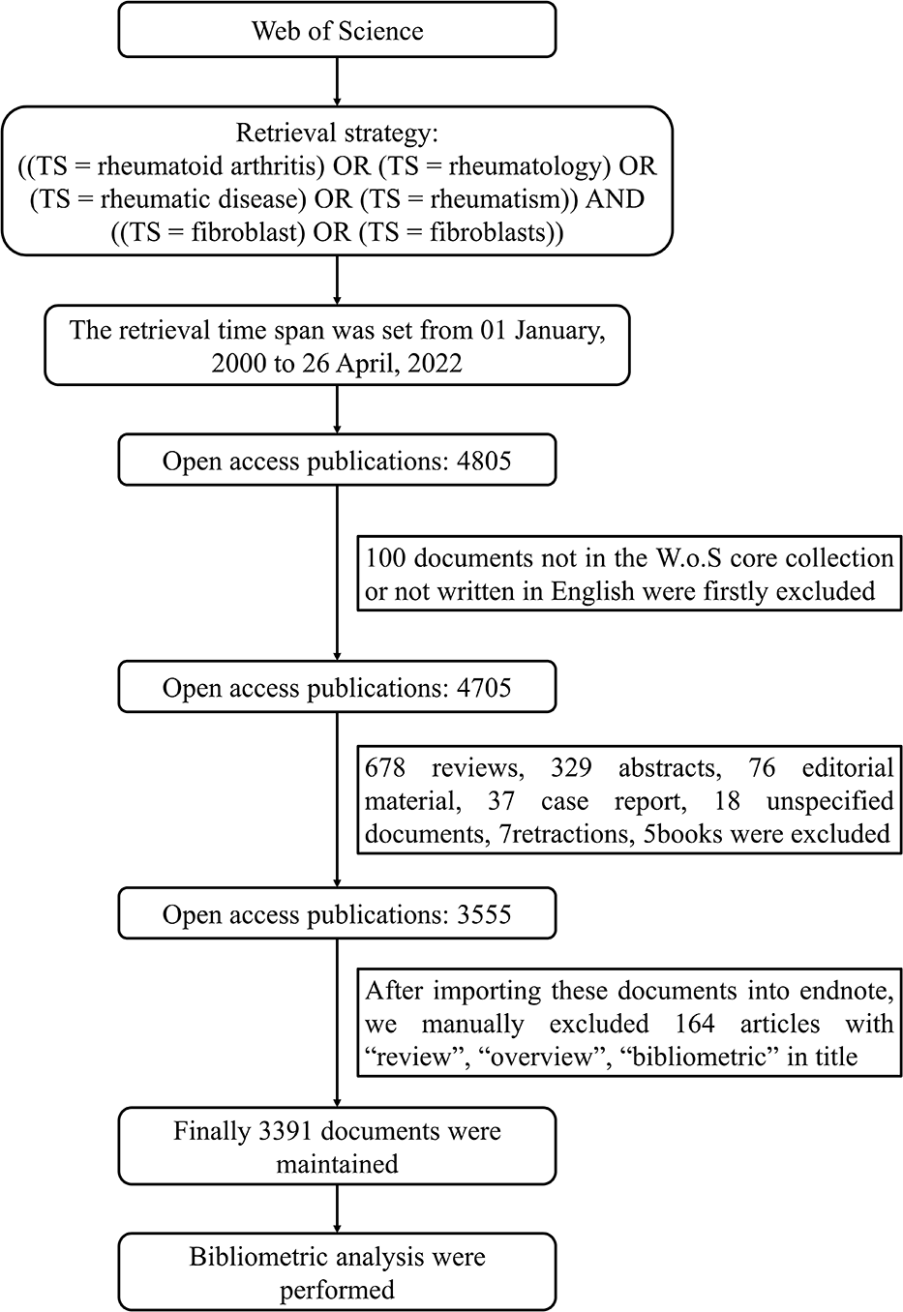
The integrated retrieval process and inclusion-exclusion criteria.

We obtained open access scientific publications from the core collection of the Web of Science (WoS) database, using the following retrieval strategy: ((TS = rheumatoid arthritis) OR (TS = rheumatology) OR (TS = rheumatic disease) OR (TS = rheumatism)) AND ((TS = fibroblast) OR (TS = fibroblasts)), year = 2000-2022, language = English. After using the W.o.S filter tool and manually removing review articles and non-research articles, 3391 studies were retrieved and exported in TXT format. The retrievals and data capture ended on April 26, 2022, and the results were imported into bibliometric tools for subsequent analysis **(** **Figure 1** **)**. Since no living creatures were involved in this study, no ethical support was required.

### 2.2 Data analysis

We mainly used the bibliometrix package in R version 4.2.0 (Institute for Statistics and Mathematics, Vienna, Austria; www.r-project.org) for quantitative analysis and visual processing of all the literature we obtained. The “Biblioshiny ()” function provided a simplified windowed interface for plotting graphs in this article (28). A series of bibliometric indicators were used to assess the output of authors, countries, institutions, and journals. The number of articles was used to assess productivity. Total citation was used to indicate the impact in the academic community, while local citation was used to assess the impact in a particular area. They were the three main dimensions to evaluate the level of research. Recently, the h-index, which means the author has h articles cited h times, has increasingly been used to evaluate the contribution of an author because it sets a threshold to combine productivity and impact (29). Also, the h-index can be extended to evaluate countries, institutions, and journals (30). In addition, CiteSpace(Version 5.6.R5) was used to assist in detecting articles with strong citation bursts and extract the keywords to help identify trend topics from different years (31). Together, VOSviewer (Version 1.6.15) helped define and plot keywords co-occurrence networks, in which the connection between two keywords indicated that they had appeared in the same article, further contributing to cluster analysis (32).

After combining the results of highly cited literature, high-frequency keywords, and keyword clustering, we manually read relevant articles to summarize the research hotspots. The development of the research was discussed based on the keyword co-occurrence network and historical direct citation network. The future research trends were deduced based on the thematic map and trend topics diagram.

## 3 Results

### 3.1 Annual publications

From 2000 to April 26, 2022, 3391 articles on RDs and fibroblasts have been published and recorded in WoS. The number of articles posted every year has been maintained at a high level since 2008, and overall, it has been rising steadily, reaching a peak in 2012 and 2020. The number of annual citations increased every year, which indicated that RDs and fibroblasts were quite popular and had significant potential in both clinical and basic research **(** **Figure S1** **)**.

### 3.2 Most productive and influential countries

**Figure 2:**
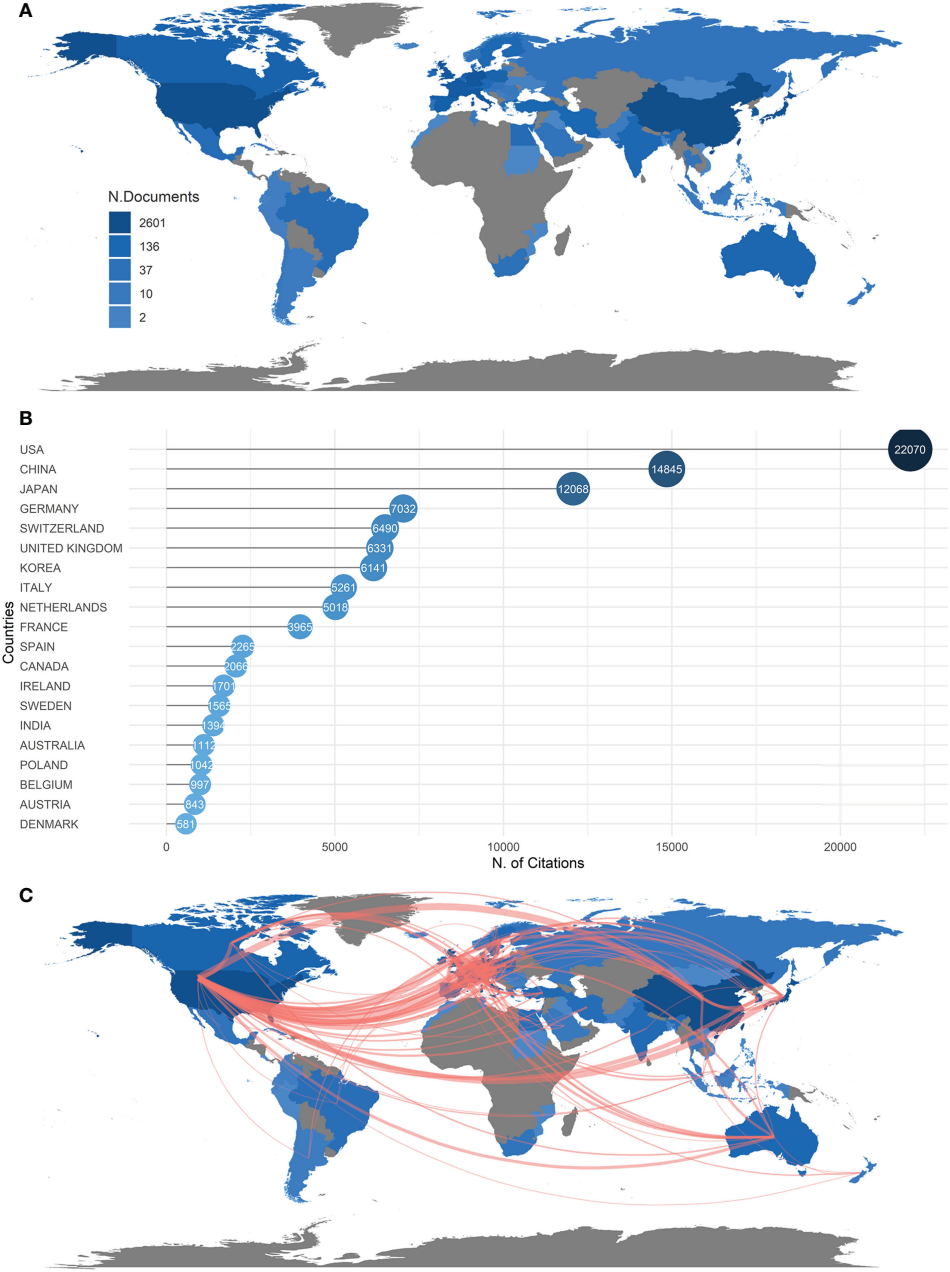
**(A)** Countries/Regions distribution of publications of RD and fibroblasts. The number of articles published by each country/region in the 21st century is indicated by the shade of color on the map. **(B)** Top 20 countries being cited the most in the 21st century. **(C)** The density of the red line represents the frequency of the collaboration work between countries.

Since 2000, 73 countries have participated in studies on RDs and fibroblasts. We used Biblioshiny to plot the numbers and distributions of publications in different countries. China is the most prolific country (n = 2601), followed by the United States (n = 2066), Japan (n = 1086), and Germany (n = 829) **(** **Figure 2A** **)**. But the United States remains the most influential country, being cited 7225 times more than China in second place **(** **Figure 2B** **)**. According to the country collaborative map, with a collaboration index of 4.86, cooperation between countries significantly contributes to the outcome. The United States and China are the two largest central connection points, linking with almost every other influential country, such as Japan, Germany, Switzerland, and Australia **(** **Figure 2C** **)**. In fact, according to the ratio of MCP (multiple country publication) to SCP (single country publication), the United States and Germany are more inclined to seek international cooperation **(** **Figure S2** **)**.

### 3.3 Most productive and influential authors and institutions

**Figure 3:**
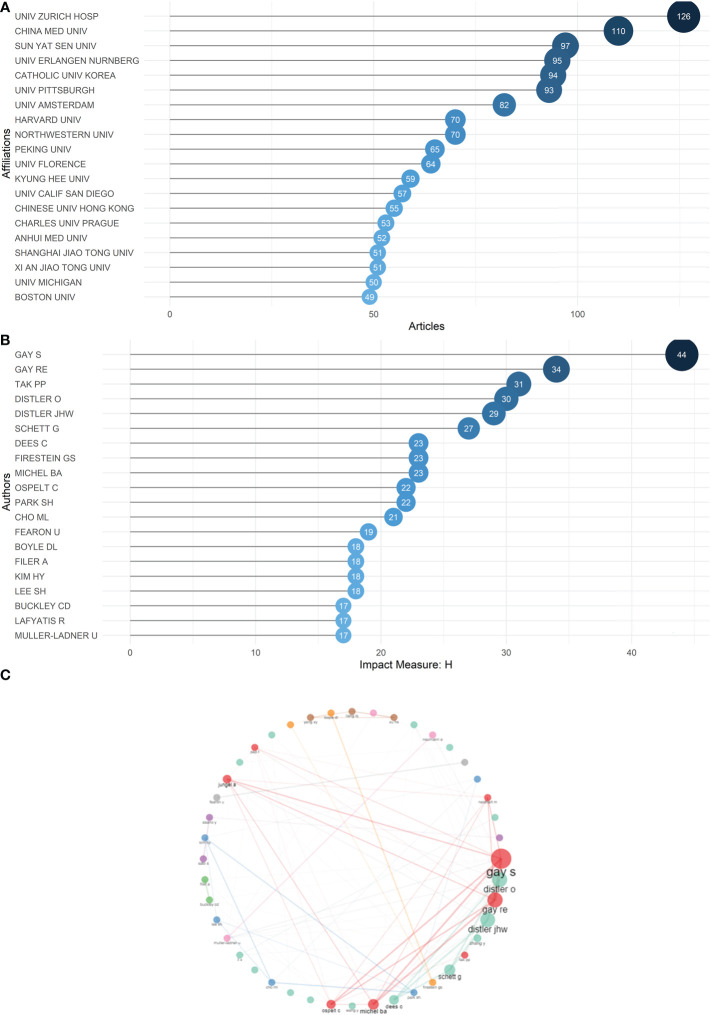
**(A)** Top 20 scientific institutions ranked by the number of articles they have supported in the 21st century. **(B)** Top 20 authors ranked by h-index in the 21st century, with a minimum threshold of 17. **(C)** Collaboration map. The size of the node represents the number of collaboration works the authors have with others, the thickness of the line connecting two authors represents the number of collaboration works between them.

A total of 16282 authors have contributed to the studies about RA and fibroblasts. Behind them are scientific institutions from all around the world, 44 of which have supported six more studies. The Center of Experimental Rheumatology at University Hospital Zürich has supported the maximum number of articles (n = 126), followed by the China Medical University (n = 110) and the Sun Yat-sen University (n = 97) **(** **Figure 3A** **)**.

To investigate the most influential authors, we used h-index as an indicator and listed authors meeting the minimum h-index threshold of 17, which meant each of them had at least 17 articles cited at least 17 times (29) **(** **Figure 3B** **)**. As indicated in **Table S1**, Steffen Gay has published 85 records with 6263 total citations, followed by Ralph E. Gay (53 records cited 4550 times), Paul P. Tak (44 records cited 2846 times), Oliver Distler (55 records cited 3672 times) and Jörg H. W. Distler (52 records cited 3095 times). These five exceptional authors dominate nearly all rankings based on different parameters, including local citations and records of publications (**Figure S3**). They also collaborate the most with other authors **(** **Figure 3C** **)**. **Figure S4** demonstrates the publications of top authors over time; they all have experienced a long track of accumulation in their fields, which gives them authority.

### 3.4 Journal analysis

Since 2000, 729 journals have published articles about RA and fibroblasts. According to Bradford’s law, based on the number of publications, 11 journals are classified as core sources (33). Together, these journals contain 1141 published articles, accounting for 33.65% of the total **(** **Figure S5** and **Table S2** **)**. Among them, Arthritis and Rheumatism, Annals of the Rheumatic Disease, and Rheumatology not only lead in the number of outputs but also have an absolute advantage in total citations and h-index **(** **Figure S6** **)**. Therefore, paying more attention to these key journals helps us better understand cutting-edge trends.

### 3.5 Most cited documents

**Figure 4:**
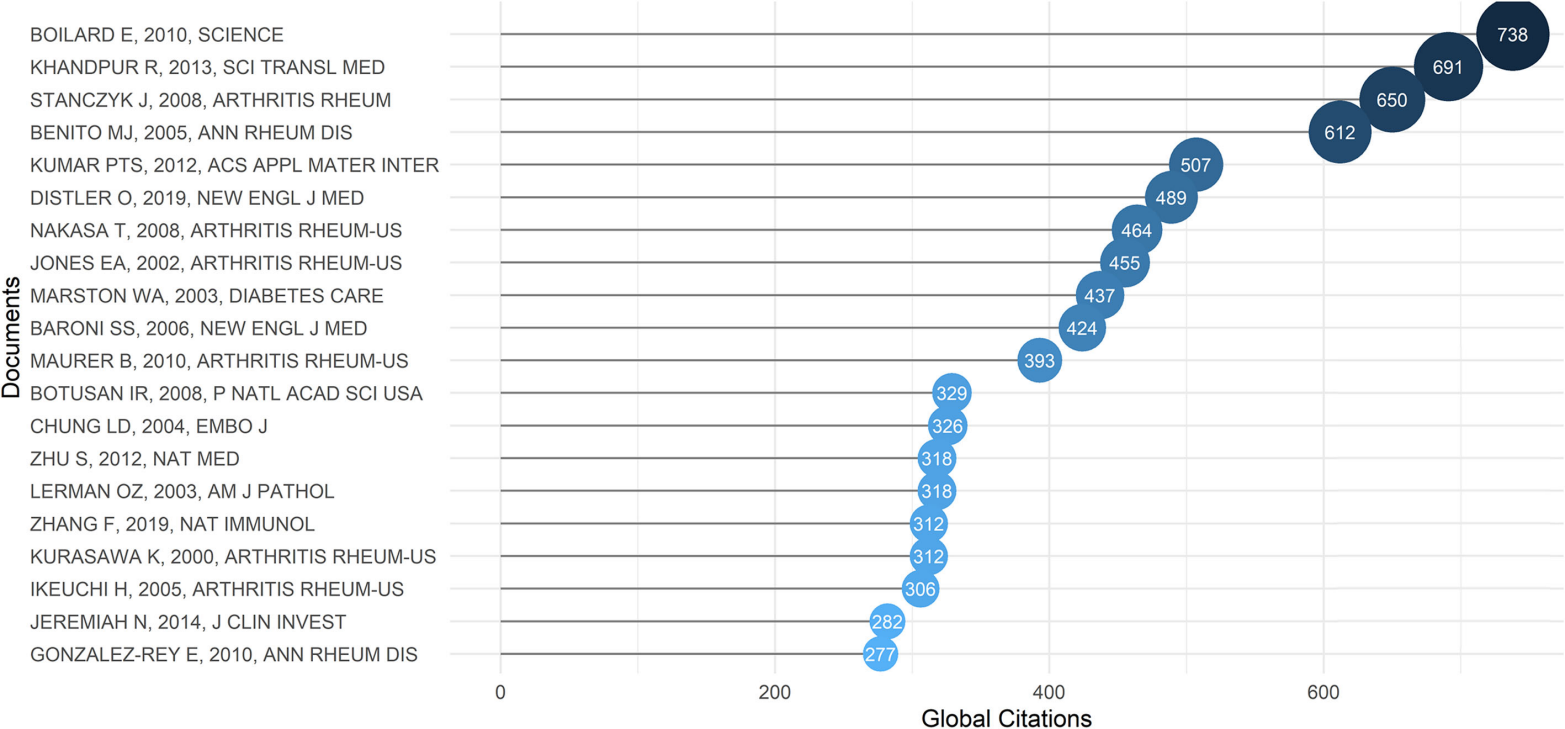
Top 20 articles ranked by total citation in the 21st century.

The table lists the 20 articles that were most cited among 3391 documents, with their journals, authors, and year of publication **(** **Figure 4** and **Table S3** **)**. Total citations are used to measure the impact or significance of a given work as well as reflect its recognition in the scientific community. Identifying highly cited papers helps locate the research or topics that have received the most attention in the 21st century.

E. Boilard et al. (34) is the most cited article. It was published in 2010, in Science, and followed by R. Khandpur et al. (35) in 2013, and J. Stanczyk et al. (36) in 2008. These three papers respectively investigated three far-reaching molecular targets: platelet microparticles, neutrophil extracellular trap (NET), and two microRNAs (miR-155 and miR-146a). They provided valuable insights for the future development of fibroblasts in RDs, and subsequent studies have further investigated multiple cytokine pathways and related intracellular signaling pathways on this basis.

Of the top 20 most cited articles, four were related to the abnormal repair of injury caused by fibroblast dysfunction in diabetes mellitus, four were related to the mechanisms of fibroblasts’ promotion of fibrosis and inflammation in SSc, and the rest were related to the inflammatory mechanism of RA, the classification of synovial cells in RA, the pathological role of FLSs, and the gene regulation of FLSs.

### 3.6 Historical direct citation network

From 2000 to 2022, there were 15 landmark papers in the fields of rheumatoid arthritis and fibroblast research **(** **Figure S7** **)**. In 2003 and 2004, two papers investigated fibroblast dysfunction in diabetes, which provided valuable ideas for the start of this field (37, 38). Since 2002, when the erosive ability of FLSs in RA was first validated (39), studies of abnormal microRNAs in fibroblasts (40), epigenetic activation of fibroblasts (41), and the newly emerging taxonomy of fibroblasts (42) have added to this network. This is also the mainstream of RDs and fibroblasts research.

### 3.7 Keywords

**Figure 5:**
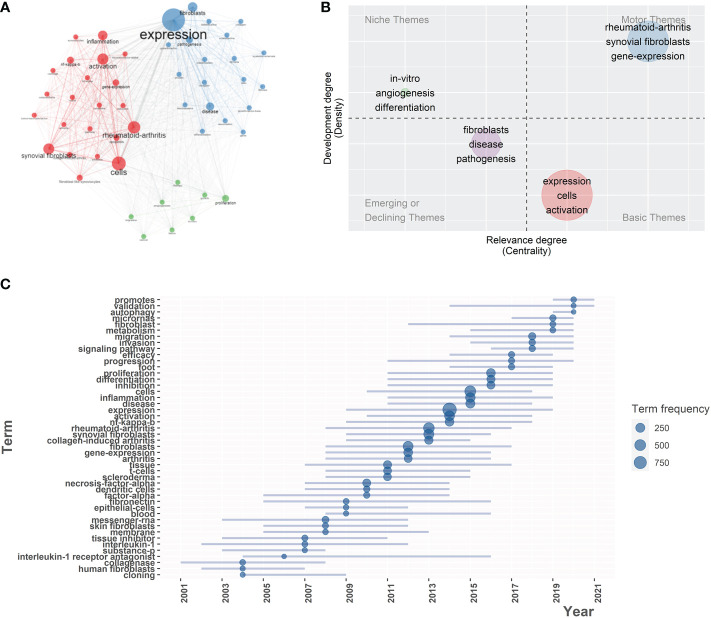
**(A)** Co-occurrence network of 50 keywords. Circle size is based on the number of occurrences. Three colors are used to indicate clusters: Blue (cluster1), Red (cluster2), Green (cluster3). **(B)** Thematic map. All articles were grouped into four clusters, each cluster corresponding to the four themes of the map according to density and centrality. Three keywords with the highest occurrence rate were displayed in each cluster. **(C)** Trend topics in the 21st century, the graph demonstrates the most frequent keywords over time. The blue dots locate at the year with the highest frequency of occurrences of this keyword.

In the keywords co-occurrence network, we analyzed the top 50 keywords that are most associated with other keywords. Each is represented by a node. The larger the node is, the more frequently it appears at the same time as other keywords. Based on the closeness of connections between nodes, keywords are divided into three clusters. Each can be condensed into a research hotspot in the fields of RDs and fibroblasts. The three clusters and their most important three keywords are listed below **(** **Figure 5A** **)**. Cluster 1 (blue): “expression”, “fibroblasts”, “disease”, and “pathogenesis”. Cluster 2 (red): “cells”, “activation”, and “rheumatoid-arthritis”. Cluster 3 (glue): “proliferation”, “*in-vitro*”, and “angiogenesis”.

As of April 26, all literature can be divided into four themes according to their subject terms **(** **Figure 5B** **)**. According to the thematic map, the characteristics of the current research subject can be visually understood, which has great reference value for the selection of future research subjects. The Y-axis of the map (Density) represents the connection intensity of basic knowledge units within a single topic. The higher the density, the higher the maturity of the theme. The X-axis of the map (Centrality) indicates the intensity of connection between a theme and other themes. The higher the centrality, the more likely it is that the theme will be at the core of all research topics. The four themes and their main keywords are listed below. Motor themes: “rheumatoid-arthritis”, “synovial fibroblasts”, and “gene-expression”. Niche themes: “*in-vitro*”, “angiogenesis”, and “differentiation”. Emerging or declining themes: “fibroblast”, “disease”, and “pathogenesis”. Basic themes: “expression”, “cells”, and “activation”.

After a detailed analysis of the trend topics, we found that the topic “expression” or “gene expression” has become more and more popular since 2008 and reached its peak in 2014. “Proliferation” and “differentiation” became popular in 2011. “Migration” came into view in 2014. The development of science and technology allowed scientists to start studying different signaling pathways in 2016 and an in-depth study of microRNA in 2017. The most recent research hotspot may be autophagy, which has become popular since 2019 **(** **Figure 5C** **)**.

### 3.8 Subgroup analysis

In order to make our study more comprehensive, we extended the time range and expanded the coverage of the retrieval formula to conduct two subgroup analyses. The retrieval formulas for the two subgroups are: 1. ((TS = rheumatoid arthritis) OR (TS = rheumatology) OR (TS = rheumatic disease) OR (TS = rheumatism)) AND ((TS = fibroblast) OR (TS = fibroblasts)), no time limitation; 2. ((TS = rheumatoid arthritis) OR (TS = Lupus Erythematosus) OR (TS = systemic sclerosis) OR (TS = psoriasis) OR (TS = Sjögren syndrome) OR (TS = rheumatology) OR (TS = rheumatic disease) OR (TS = rheumatism) OR (TS = inflammatory bowel disease) OR (TS = enteropathic arthritis) OR (TS = ANCA associated vasculitis) OR (TS = dermatomyositis) OR (TS = polymyositis)) AND ((TS = fibroblast) OR (TS = fibroblasts)), no time limitation. The retrievals and data capture ended on January 3, 2023. We listed the annual publications, the most influential countries, authors **(** **Figure S8** **)**, institutions, and journals **(** **Figure S9** **)**. We also showed the most cited articles in **Tables S4**, **S5**, and finally, we plotted the trend topics **(** **Figure S10** **)**. After we extended the time span and expanded the coverage of the formula, we found that the countries, authors, institutions, and journals mentioned above still have dominant power, but many highly cited articles from before 2000 appeared in the list. Although our study mainly focused on the relationship between the development of technology and the research results since 2000, the following discussion still drew on these classic literatures. Furthermore, although fibroblasts also influence other RDs such as SLE and psoriasis, most retrieval results are focused on RA and SSc due to the high incidence and decisive role of fibroblasts in these two diseases.

## 4 Discussion

We summarized the development of research on fibroblasts in RDs by combining the historical direct citation network, keywords co-occurrence network, thematic map, and trend topics.

Two main diseases of RDs, systemic sclerosis (SSc) and rheumatoid arthritis (RA), are closely related to fibroblasts in their pathogenesis and disease progression. In SSc, fibroblasts are overactivated and succeed in escaping apoptosis, resulting in excessive fibrosis, sclerosis, atrophy, and eventual dysfunction of the skin and internal organs (43). In RA, fibroblasts in the sub-lining of the joint synovium differentiate into fibroblast-like synoviocytes (FLSs). In addition to having the same overaction and anti-apoptotic ability as fibroblasts in SSc, they can also recruit inflammatory cells (44), synthesize destructive proteins (45), activate osteoclasts (46), and migrate throughout the body (47), which plays an important role in arthritis and bone erosion. Although fibroblasts in SSc were discovered 10 years earlier than FLSs (1972 and 1982) (16, 48), due to the limitations of sequencing technology, the research progress in SSc fibroblasts and FLSs have been synchronized since 2000. FLSs have more pathological mechanisms to explore than SSc fibroblasts, and the incidence of RA is much higher than that of SSc. Therefore, this study will mainly focus on the development of FLSs.

We discovered that fibroblasts in RDs is a fairly mature research field through bibliometric analysis. Since the next-generation sequencing technology began to be applied to the human genome in 2008, the annual publications in this field have been increasing every year, reaching the peak in 2012. But the number of publications has not increased since then. It wasn’t until sequencing technology reached microRNA in 2017 that the field was revived. From an international perspective, the economically developed countries such as the United States, China, Japan, Germany, and so on are among the top in terms of publication output and influence. This may be because economic development can make researchers in these countries access emerging technologies earlier, such as next-generation sequencing or single-cell sequencing. We have listed the five most influential authors in 3.3, the most influential journals in 3.4, and the most cited articles in 3.5. We recommend that researchers looking to get into this field pay attention to what the teams of these authors are doing, the latest publications of these journals, and carefully read the papers we listed in **Table S3**.

The historical direct citation network lines two main networks, consisting of 15 landmark articles. Together with the trend topics, it helps us explore the changes in the content of studies on fibroblasts in RDs. In 2003, O. Z. Lerman et al. demonstrated that in diabetes, fibroblasts cannot up-regulate VEGF (vascular endothelial growth factor) in time under hypoxia, resulting in slow wound healing (37). In 2004, S. K. Han et al. found that using fresh human fibroblast allografts could treat diabetic foot ulcers (38). In the study of diabetes, the transcription factor HIF promotes angiogenesis and the recruitment of inflammatory cells by activating the transcription of the angiogenic gene VEGF (vascular endothelial growth factor), which plays a guiding role in abnormal angiogenesis and tissue hyperplasia in RDs.

At the beginning, T. C. A. Tolboom et al. found that FLSs enhanced their erosive ability *in vitro* by expressing matrix metalloproteinases (MMP) (39), and K. W. Kim et al. verified that FLSs promoted the activation and differentiation of osteoclasts in synovial tissues through high expression of Receptor Activator for NF-κB Ligand (RANKL) (49). MMP and RANKL are essential for FLSs to cause joint damage, which are also trending topics at the beginning of the century, and will continue to be emphasized in later studies. There are different types of MMP that decompose collagen. For example, MMP-1 is released from FLSs as a proenzyme. Then MMP-3 activates the proenzyme into collagenase, which breaks down type IV collagen (50). MMP-13 directly secreted by FLSs has affinity for type II collagen and is adhered to cartilage, so it may be the main MMP involved in joint destruction (51). In addition to directly causing joint damage, FLSs can also destroy bone by activating osteoclasts through the synovial cytokine RANKL, which promotes monocyte migration and aggregation of osteoclast precursors in the synovium (52). In addition, FLSs can also secrete IL-6 to recruit more inflammatory cells to the lesion site (53).

In 2005, F. Brentano et al. suggested that RNA released by necrotic synovial cells may be the endogenous ligand of Toll-like receptor-3 (TLR-3), thereby activating the pro-inflammatory gene expression of FLSs (54). In 2007, K. W. Kim et al. investigated the effect of the TLR related signaling pathway in FLSs on RANKL expression (49). Since then, researchers have conducted extensive studies on cytokines and signaling pathways that regulate FLS function. TNF-α secreted by macrophages promotes the synthesis of IL-1β by FLSs. TNF-α and IL -1β have both been found to overexpress collagenase, MMP-13, and prostaglandin in FLSs (55, 56). TGF-β, as a member of the growth factor family, can inhibit the secretion of MMP by FLSs and induce the differentiation of FLSs into alpha-smooth muscle actin-positive myofibroblasts to repair damaged joints (57). However, both in RA and SSc, it is precisely the positive regulation of TGF-β that increases the pathological proliferation of fibroblasts and the ability of migration and invasion (58). Cadherin-11, which mediates cellular adhesion, has also been found to promote the self-aggregation and migration of FLSs to different articular cartilages (59). Cytokines bind to specific receptors on the FLS membrane and lead to phenotypes through a series of intracellular signaling pathways. There are too many cytokines that can influence the FLS phenotype, but ultimately the synthesis of pathogenic proteins such as MMP and RANKL can be achieved by inhibiting some of the related signaling pathways. A variety of cytokines may eventually increase the invasiveness and destructiveness of FLSs by activating transcription factors such as NF-κB (nuclear factor kappa B) and MAPK (mitogen-activated protein kinase), also representing two important intracellular signaling pathways. For example, IL-1 induces the gene expression of collagenase through the MAPK signaling pathway, and JNK is a key signal molecule in the MAPK signaling pathway (60). Therefore, therapy targeting JNK may have decent efficacy. Similar patterns have been used for RANKL, which increases NF-κB to activate osteoblasts. TGF-β was recently discovered to induce EMT (epithelial-mesenchymal transition), a key mechanism of cancer cell migration and invasion, by activating the Smad2/3 signaling pathway in FLSs (58).

In 2008, T. Nakasa et al. proposed that miR-146 expression is increased in RA synovial tissues after stimulation with TNF-α and IL-1β, which is the great innovation brought to this field by next-generation sequencing (40). Since then, researchers have found more molecules regulating gene expression that are associated with known FLS phenotypes, like microRNAs and lncRNAs. For example, miR155 inhibits the expression of MMP-1 and MMP-3 (36), miR-124a inhibits the proliferation of FLSs (61), and miR-34* promotes FLS resistance to apoptosis through its low expression (62). There are also some small molecules that can directly kill abnormal fibroblasts, such as miR-613, which can induce apoptosis of FLSs by targeting DKK1 expression (63) and miR-29a, which can induce apoptosis of dermal fibroblasts (64). In 2009, E. Karouzakis et al. found that the activation of FLSs may be related to DNA hypomethylation (65). In 2011, J. Stanczyk et al. found that the expression of miR-203 in FLSs is regulated in a methylation-dependent manner, and the increased level of miR-203 promotes the activation of FLSs through the NF -κB pathway (41). Epigenetic inheritance has since become a popular frontier of research. Abnormalities in DNA methylation, histone modification, and microRNA regulation affect gene expression and transcription in fibroblasts and help better classify fibroblasts (66). With the development of the lncRNA detection probe chip, lncRNAs gradually come into view and inherit the popularity of microRNAs (67), providing more material for transcriptome analysis. However, there is only a little research in this area, which may be a feasible direction of development.

With the widespread use of single-cell sequencing technology (scRNA-Seq) since 2013, we have improved our ability to examine gene expression at the single-cell level and to find different fibroblast subpopulations in various organs and tissues. It was found in 2018 that fibroblasts in the synovial lining mediate bone and cartilage damage, while fibroblasts in the sub-lining of the synovium mediate inflammation and promote the persistence of inflammatory arthritis but have little effect on bone and cartilage (68). Combining multiple bioanalytical techniques, including bulk RNA sequencing, single-cell RNA sequencing, mass cytometry, and flow cytometry, researchers were able to classify the cells more precisely. For example, a study conducted in 2019 of synovial cells found that IL-6 expression could be attributed to THY1 (CD90) (+) HLA-DRA (hi) fibroblasts (69). Fap α (+) THY1 (–) fibroblasts express on their membrane and secrete high levels of RANKL (70).

Multiple cytokines (TNF-α, TGF-β, IL-1, and IL-6) in RDs bind to the receptors on the surface of fibroblasts and induce the expression of pathogenic genes through intracellular signaling pathways (mainly MAPK and NF-κB), so that fibroblasts oversecrete collagen or destructive substances like MMP and RANKL. The processes of cell proliferation, synthesis, secretion, and apoptosis are all affected by epigenetic inheritance through DNA methylation, histone modification, and microRNA regulation. With the progress of sequencing technology, lncRNA has become more and more popular in transcriptome analysis in recent years.

An analysis of keywords can tell us the main research areas and the direction of future development in RDs and fibroblasts. The keywords co-occurrence network divides all high-frequency keywords into three clusters.

Cluster 1 (blue): regulation of fibroblasts. This cluster focuses more on the related cytokines and pathways that affect fibroblast function and apoptosis in pathological tissues. Among them, tumor necrosis factor-α (TNF-α) is the most studied cytokine and nuclear transcription factor-κB (NF- κB) represents the most popular signaling pathway. TNF-α is primarily secreted by activated T lymphocytes, and members of the TNF superfamily act as activator of NF-κB receptor. NF-κB is a class of key nuclear transcription factors directly involved in lymphocyte development and activation, stress response, and apoptosis of fibroblasts. NF-κB is involved in the transcription of more than 60 genes, such as those involved in cell adhesion, immune stimulation, apoptosis, chemotaxis of inflammatory cells, and cell differentiation, many of which are associated with the pathological features of fibroblasts.

Cluster 2 (red): activation of different subsets of fibroblasts. This cluster focuses more on how normal fibroblasts differentiate into different subsets through cytomics and transcriptomics in order to explore and explain the heterogeneity of symptoms and pathological changes in patients with RDs, hence providing ideas for personalized treatment. TGF-β (transforming growth factor-β) used to hold a dominant position due to its ability to mediate the transformation of normal fibroblasts into invasive fibroblasts. However, with the continuous popularization and optimization of cell sequencing technology, more studies focus on the analysis of small molecules such as miRNA and lncRNA that play a more specific role in the phenotypic change of fibroblasts. However, as almost all the key RNAs discovered have not been verified by translational medicine, it is difficult to summarize the recognized research hotspot.

Cluster 3 (green): *in-vitro* validation of fibroblasts. This cluster focuses on *in-vitro* validation tests. The studies are committed to reproducing and verifying the pathological changes and related signaling pathways and regulatory molecules of fibroblasts *in vitro*. They not only provide raw materials for subsequent research but also lay the foundation for the clinical transformation of the above-mentioned theories.

In general, RDs and fibroblast research can be divided into three broad categories: the role of TNF and NF-κB in fibroblast functional regulation and apoptosis; the role of TGF-β and microRNAs in normal fibroblast differentiation and activation into different subsets; and *in-vitro* validation and clinical transformation of the aforementioned discoveries. This corresponds to the three themes of the thematic map, in which researchers can conduct research. We analyzed their advantages and disadvantages below for reference.

Motor themes represent the mainstream of research in this field, with strong centrality and density. Since the sequencing breakthrough in 2008, scientists have conducted a large number of studies on gene expression regulating the function of fibroblasts with different phenotypes. We believe research in this area is relatively mature, and it is easy to gain more experience. However, the competition is also fierce, so it is difficult to obtain breakthrough progress.

Basic themes are themes of low maturity in this field with strong centrality but weak density. They focus more on the activation mechanisms of different fibroblasts. These themes are recommended the most because there is still a lot to be clarified about the activation mechanisms of different types of fibroblasts. As long as the activation mechanism can be clarified and drugs can be developed to inhibit the main activation pathway, RD can also be prevented in a pre-clinical phase, which endows research in this direction with direct clinical significance.

Both Motor themes and Basic themes have been well explored with transcriptome analysis and involve many epigenetic studies. Therefore, future development will also be closely related to the sequencing technology, such as the lncRNA detection probe chip.

Niche themes are isolated themes of high maturity in this field with weak centrality but strong density. They are dedicated to *in-vitro* validation of the proposed theory. This is of great value to existing proteome, genome, and transcriptome analyses for clinical transformation. For example, Artesunate (ART) inhibits TNF-α-induced IL-1β, IL-6, and IL-8, hypoxia induced HIF-1α (hypoxia-inducible factor-1α) expression (71), as well as the secretion of VEGF (vascular endothelial growth factor) and IL-8 (72). As animal models were established, ART inhibited the NF-κB and MAPK pathways to inhibit the action of pro-inflammatory cytokines and the activity of MMP-9 in mouse models (73). After more animal trials, the drug may be ready for clinical trials, providing a new way to treat RA. Since there are so many molecular targets that have been discovered, the research in this area is of great significance and possibility for the clinical transformation of basic research, but it may need more work and resources.

Besides, the thematic map also gave the 4^th^ theme, “emerging or declining themes”. However, after analyzing it with clinical knowledge, it’s a theme that is declining in popularity. As it is known that fibroblasts can recruit inflammatory cells, promote pathological fibrosis, synthesize destructive proteins, activate osteoclasts, and migrate throughout the body, we see little value in looking for more evidence that fibroblasts contribute to the pathogenesis of RD.

After analyzing the trend topics, we found a close relationship between the study of fibroblasts in RD and the development of sequencing technology. In 2008, the first application of next-generation sequencing technology to the human genome was announced, allowing a human genome to be sequenced in several weeks at a much lower cost (74). In the same year, RNA sequencing technology pioneered transcriptomics, bridging the gap between genomics and proteomics. Accordingly, since 2008, research on gene expression has become increasingly popular and reached its peak in 2014. Hence, most researchers have focused on looking for and inhibiting the signaling pathways of pathologic fibroblast activation, proliferation, and differentiation to enhance the efficacy of drugs. In 2009, Nature Methods published the first full transcriptome sequencing study of a single mouse blastomere, bringing single-cell sequencing technology to the forefront. Single-cell sequencing was later named “Technology of the Year” by Nature Methods in 2013, saying it would transform many areas of biology and medicine (75). Since then, researchers have been able to type the fibroblasts in more detail and focus on a range of signaling pathways that make certain subsets of fibroblasts migratory and invasive. Since 2020, as many molecular mechanisms have become clear and *in vitro* validation experiments have been carried out, researchers have been committed to verifying and looking for the most reliable therapeutic targets (76, 77). With the understanding of the fibroblast pathologic process, how to regulate fibroblast autophagy has become an emerging hot topic. For example, knock down of miR-218-5p could regulate the autophagy of FLSs (78) and targeting PGC-1α prevents abnormal autophagy, inhibits fibroblast activation and tissue fibrosis in SSc (79). To sum up, the understanding of fibroblasts in RDs is becoming comprehensive and is gradually being transformed into clinical practice. It is believed that clinical results will bring benefits to patients in the near future.

## Data availability statement

Publicly available datasets were analyzed in this study. This data can be found here: The datasets generated are available in the Web of Science™ (WOS, http://www.webofknowledge.com). The data sets we have downloaded and used for analysis can be found in supplementary materials.

## Author contributions

Conception/design: RH, MJ, YFL, YWL, MZ, PY, SX, SW, HZ, XZ,SC, BL, YY, ZH, XL, SJ. Collection and/or assembly of data: RH, MJ, YFL, YWL, MZ, PY, SX, SW, HZ, XZ, SC, BL, YY, ZH, XL, SJ. Data analysis and interpretation: RH, MJ, YFL, YWL, MZ, PY, SX, SW, HZ, XZ, SC, BL, YY, ZH, XL, SJ. Manuscript writing and Final approval of manuscript: RH, MJ, YFL, YWL, MZ, PY, SX, SW, HZ, XZ, SC, BL, YY, ZH, XL, SJ. All authors contributed to the article and approved the submitted version.
