# Supplementary material for: Global trends in research of fibroblasts associated with rheumatoid diseases in the 21st century: A bibliometric analysis
Source: Front Immunol. 2023 Feb 10;14:1098977. doi: 10.3389/fimmu.2023.1098977 (PMC9950622; doi:10.3389/fimmu.2023.1098977)
Supplement: Supplementary file 1 [file DataSheet_1.docx]

**Supplementary Materials**


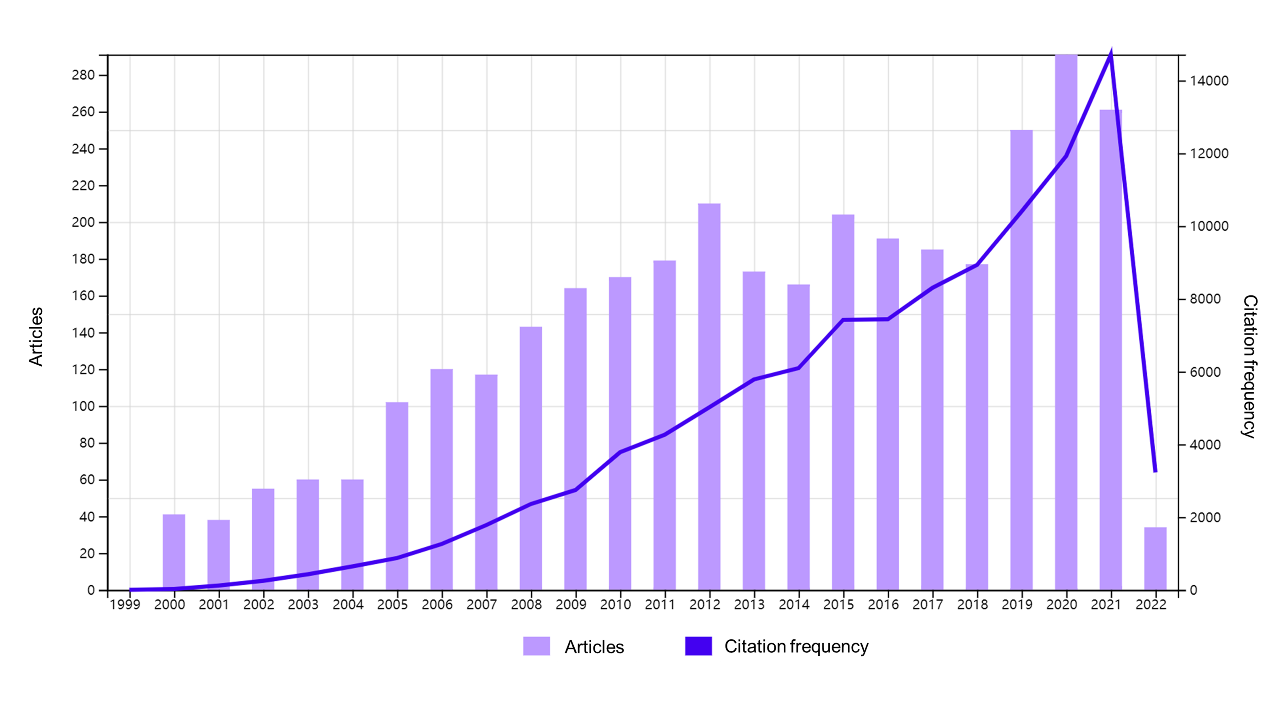


**Figure S1.** Global scientific production of RDs and fibroblasts from 2000 to 2022 annually.


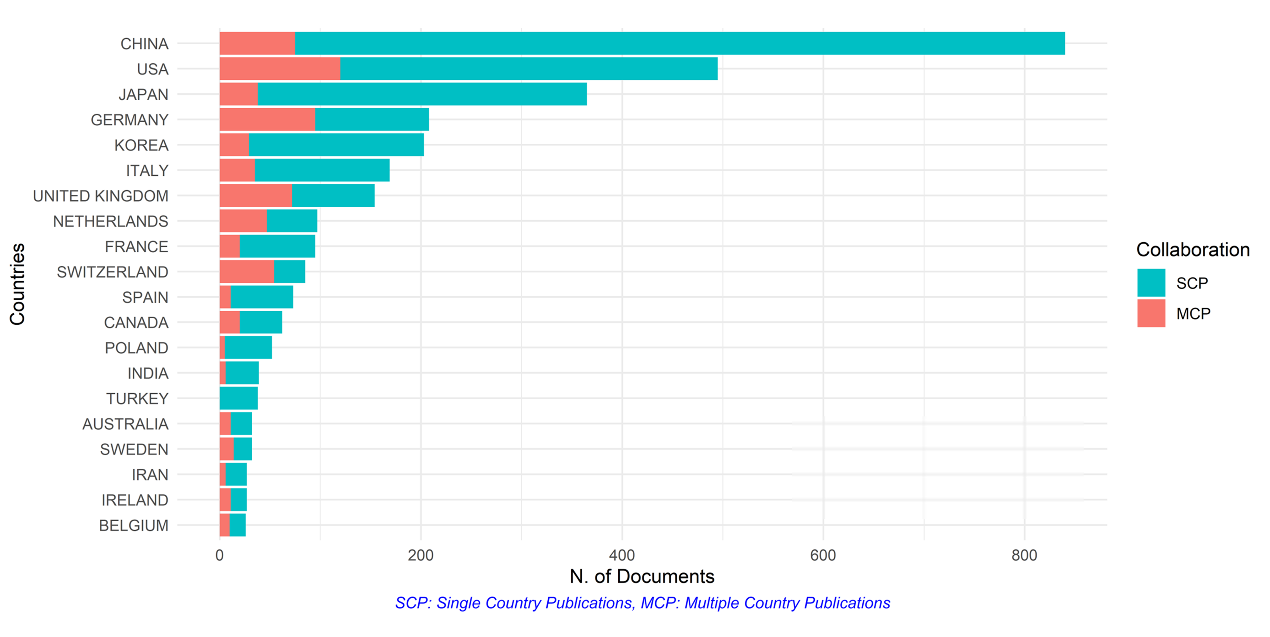


**Figure S2.** The histogram illustrates the proportion of Multiple countries publication (MCP) and single country publication (SCP) in the most productive countries.


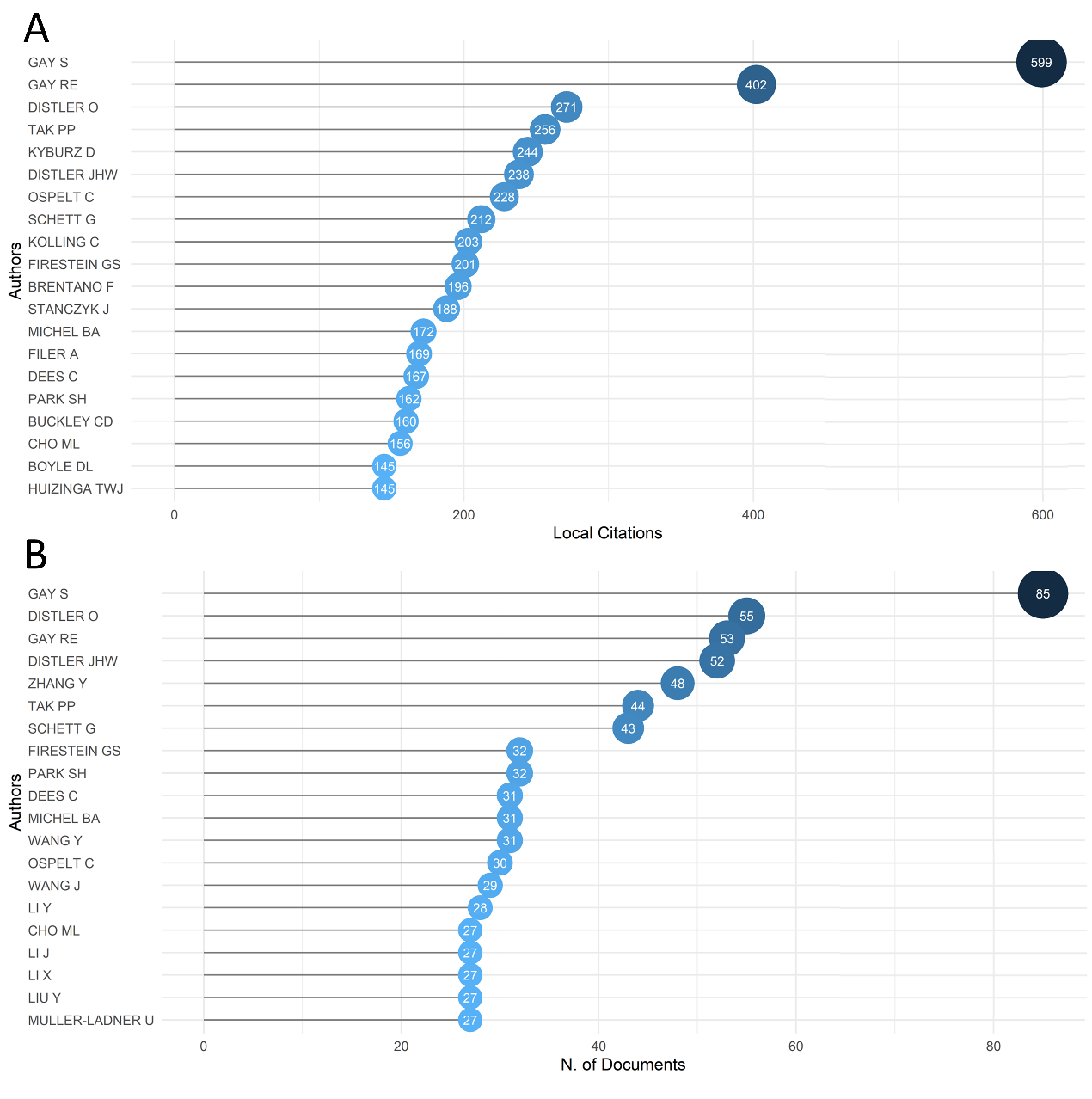


**Figure S3. (A)** Top 20 authors ranked by the number of local citations in the 21st century. **(B)** Top 20 authors ranked by records of publications in the 21st century.


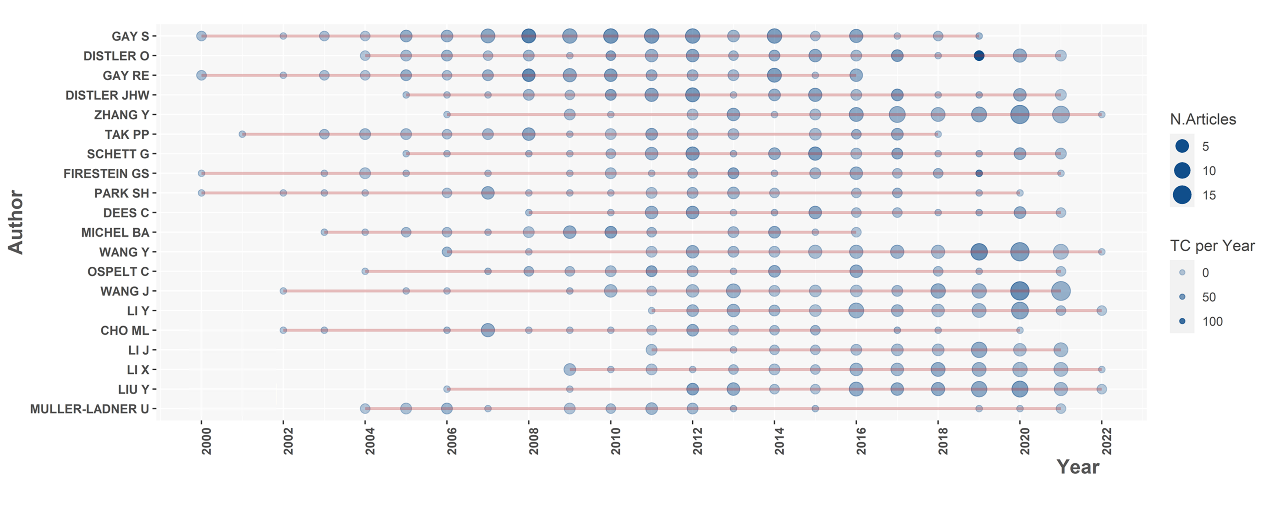


**Figure S4.** Top-author’s production over time. The size of the nodes was related to the number of documents and the color intensity was related to the total citations per year.


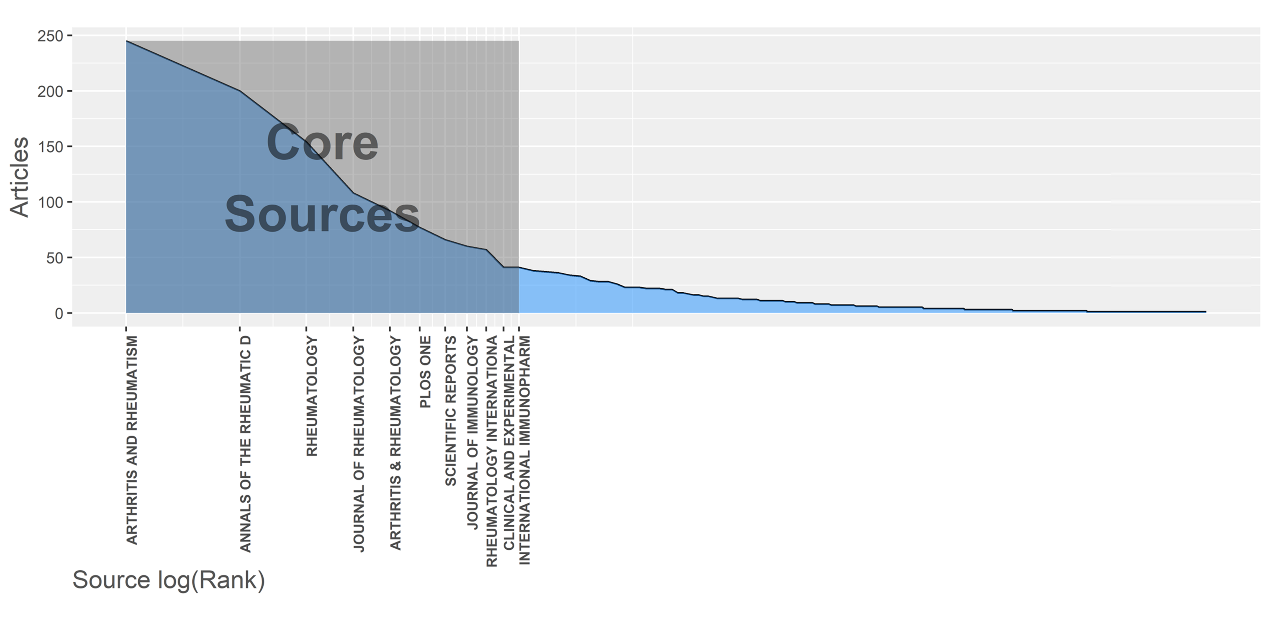


**Figure S5.** Bradford’s law serves as an indicator for the dispersion of scientific publications. Journals in the core sources published about a third of all articles.


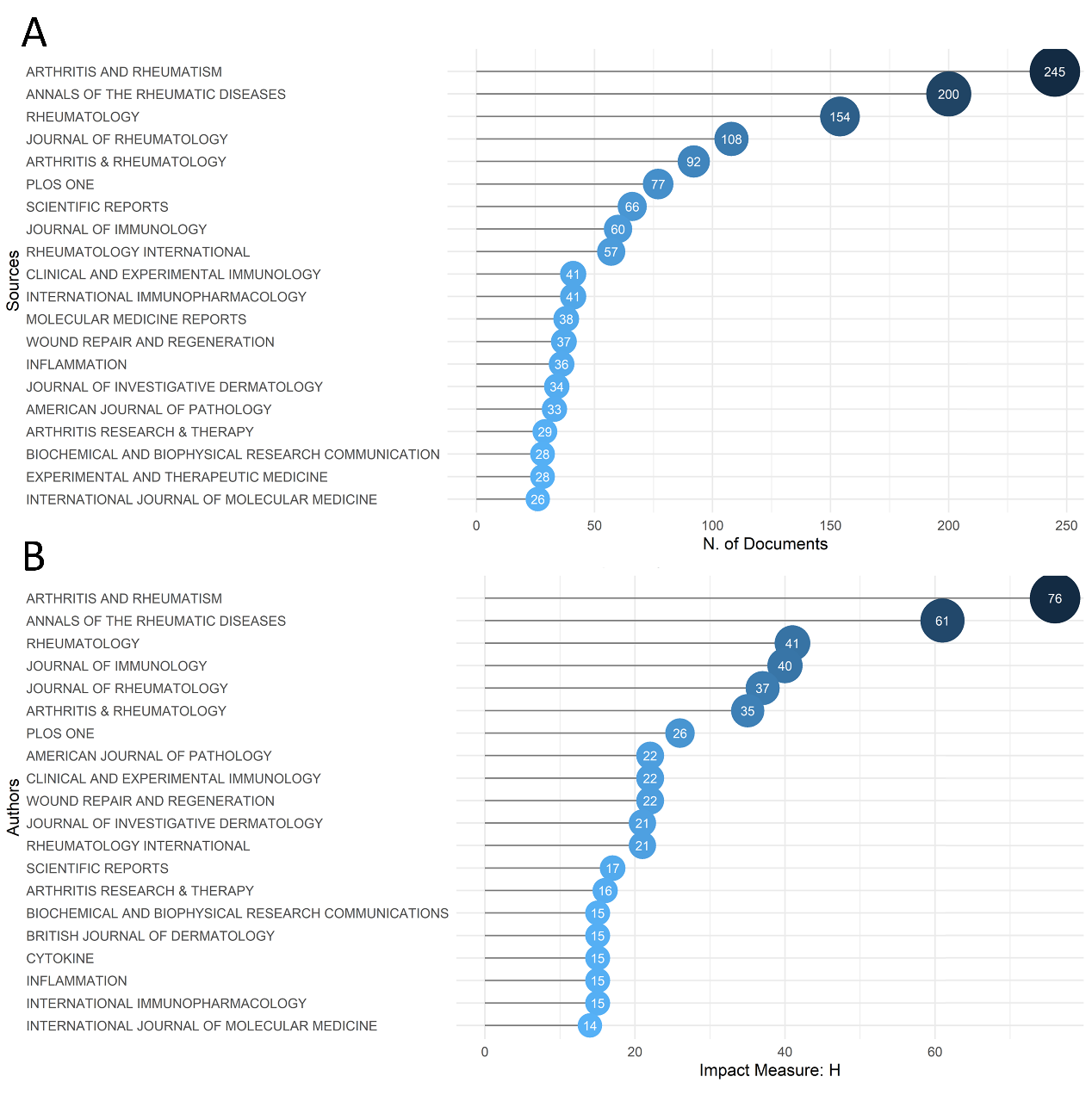


**Figure S6. (A)** Top 20 journals published the most articles in the 21^st^ century. **(B)** top 20 journals ranked by h-index in the 21^st^ century.


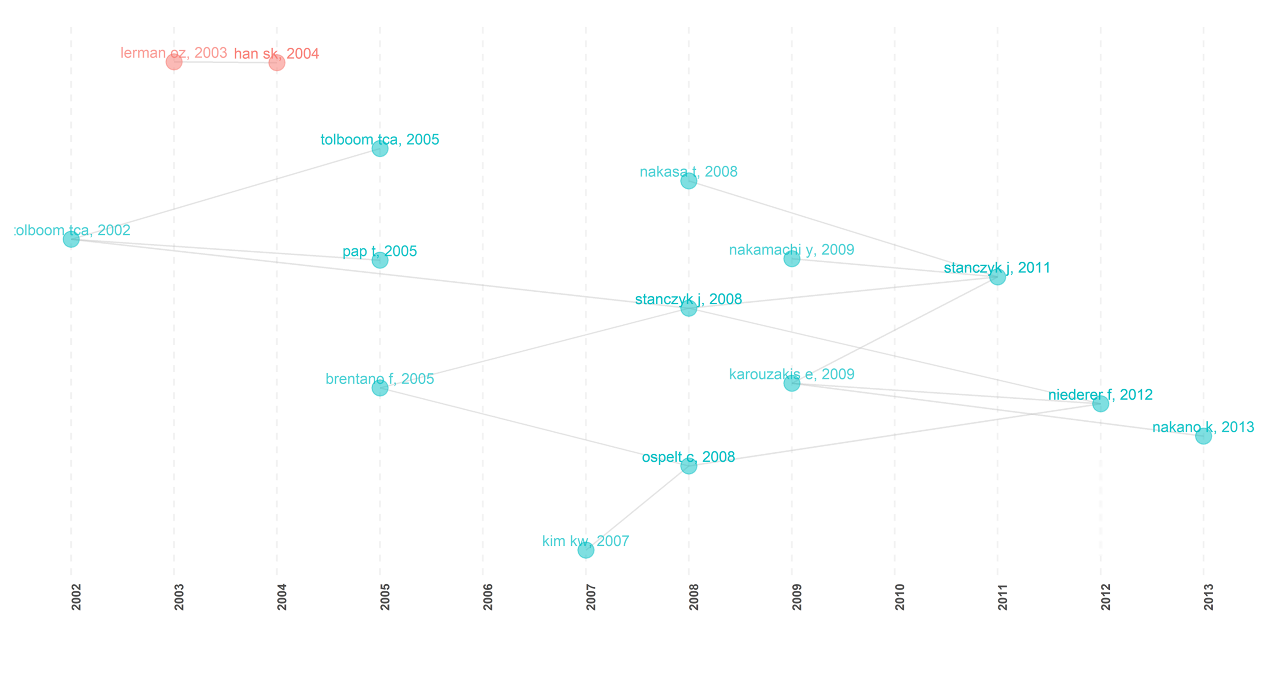


**Figure S7.** Historical direct citation network of fibroblasts in RD. Each node represents a historical landmark article, and the references between them are indicated by lines.


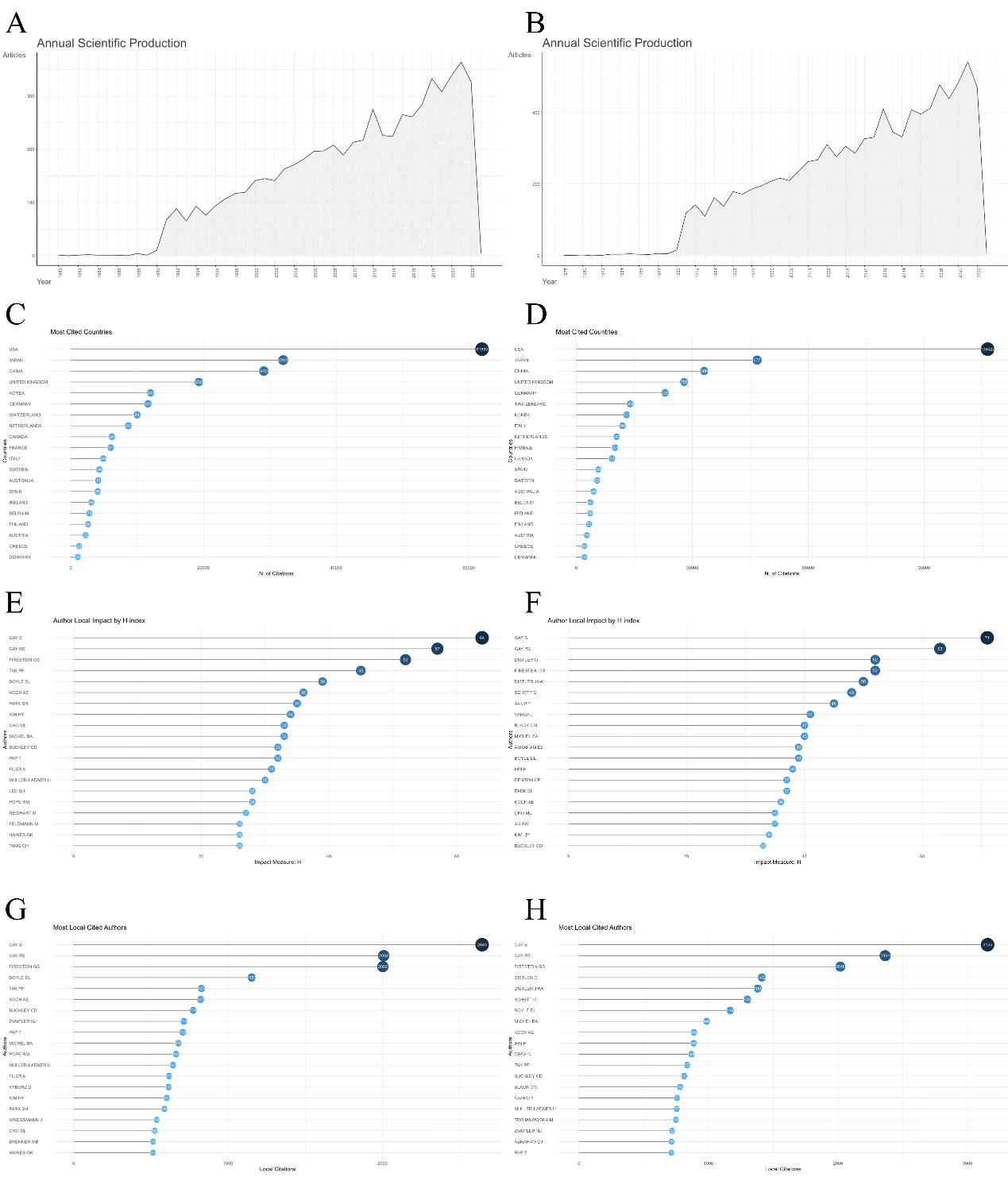


**Figure S8.** **(A)** Global scientific production of subgroup 1. **(B)** Global scientific production of subgroup 2. **(C)** Top 20 countries being cited the most in subgroup 1. **(D)** Top 20 countries being cited the most in subgroup 2. **(E)** Top 20 authors ranked by h-index in subgroup 1. **(F)** Top 20 authors ranked by h-index in subgroup 2. **(G)** Top 20 authors ranked by the number of local citations in subgroup 1. **(H)** Top 20 authors ranked by the number of local citations in subgroup 2.


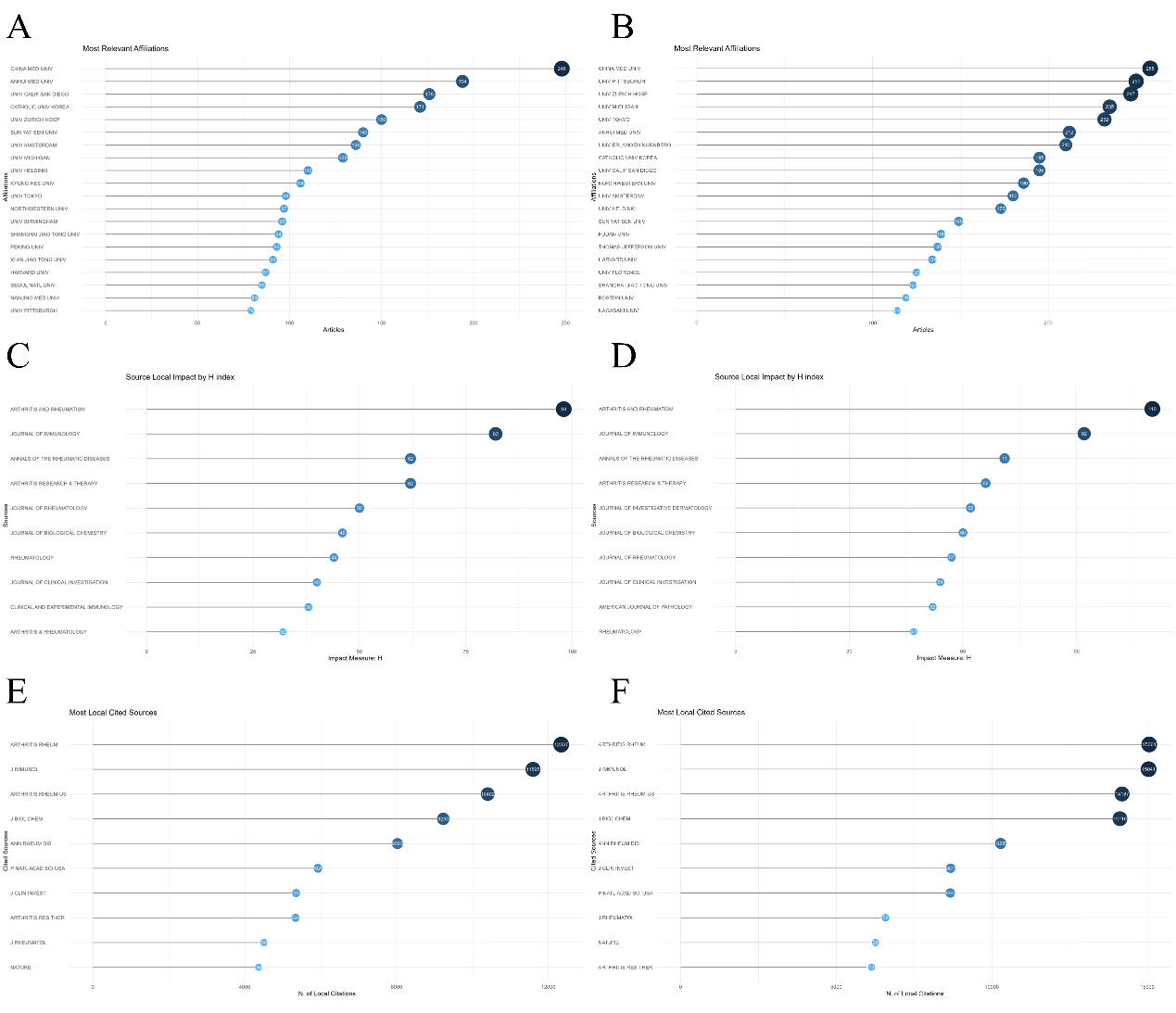


**Figure S9. (A)** Top 20 scientific institutions ranked by the number of articles they have supported in subgroup 1. **(B)** Top 20 scientific institutions ranked by the number of articles they have supported in subgroup 2. **(C)** Top 10 journals ranked by h-index in subgroup 1. **(D)** Top 10 journals ranked by h-index in subgroup 2. **(E)** Top 10 journals ranked by the number of local citations in subgroup 1. **(F)** Top 10 journals ranked by the number of local citations in subgroup 2.


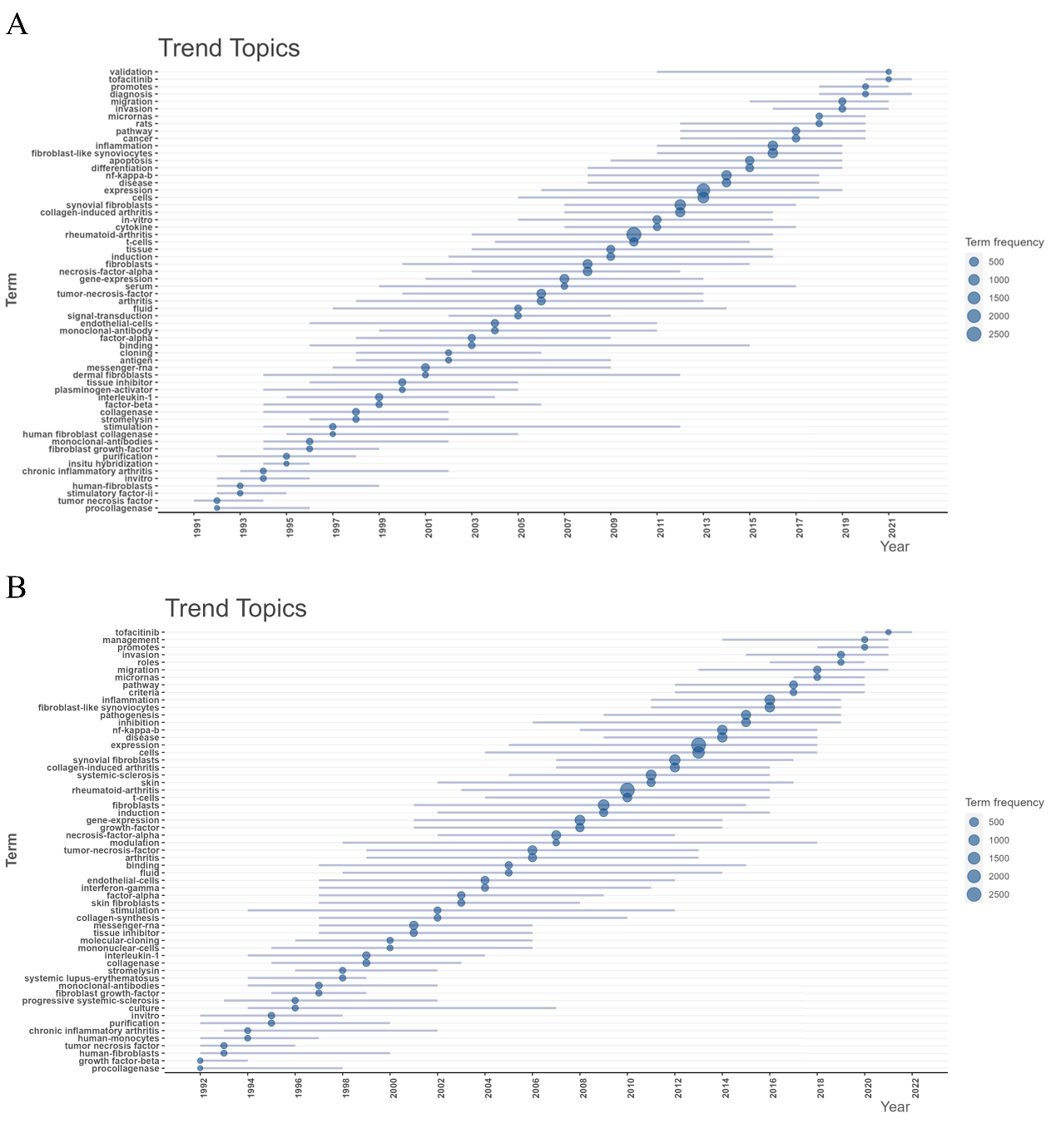


**Figure S10. (A)** Trend topics of subgroup 1, the graph demonstrates the most frequent keywords over time. The blue dots locate at the year with the highest frequency of occurrences of this keyword. **(B)** Trend topics of subgroup 2.


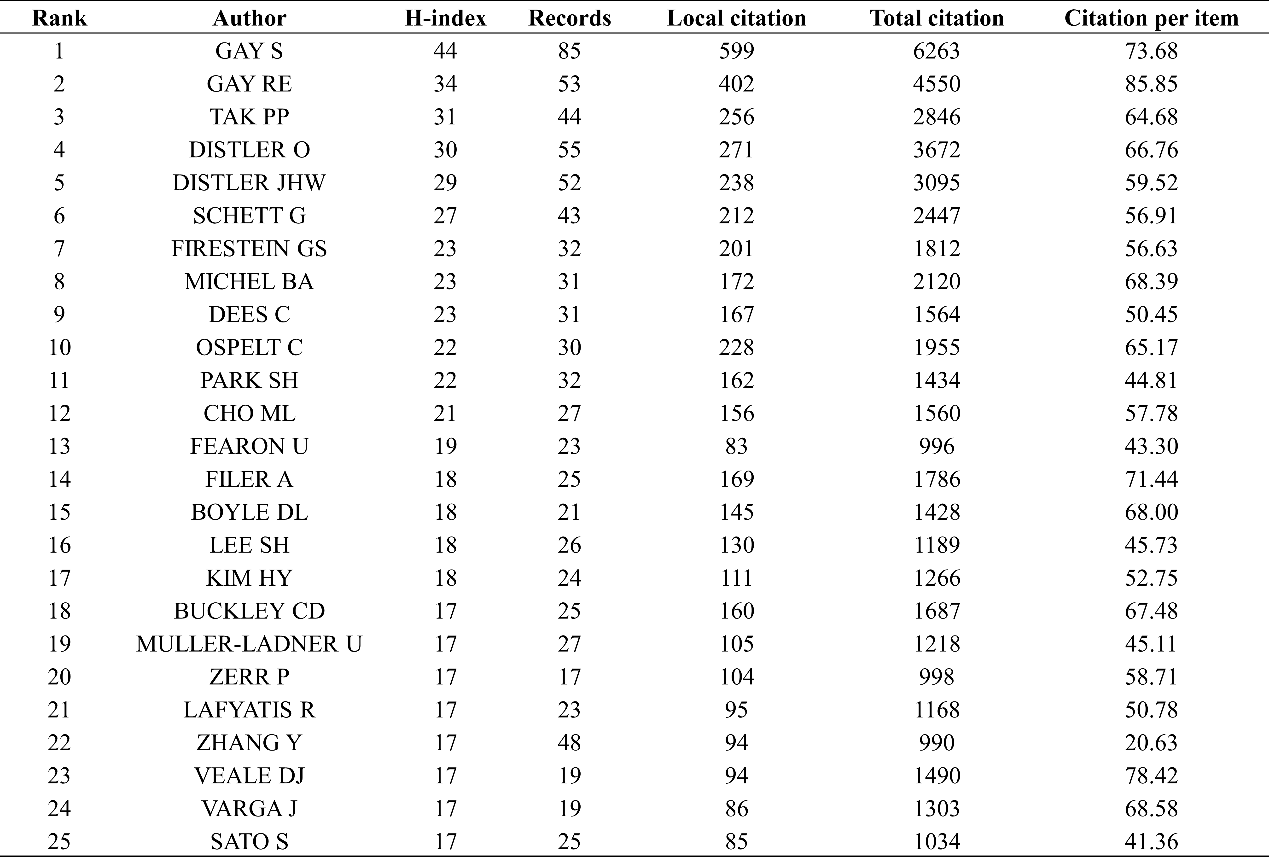


**Table S1.** Top 25 authors ranked by h-index.


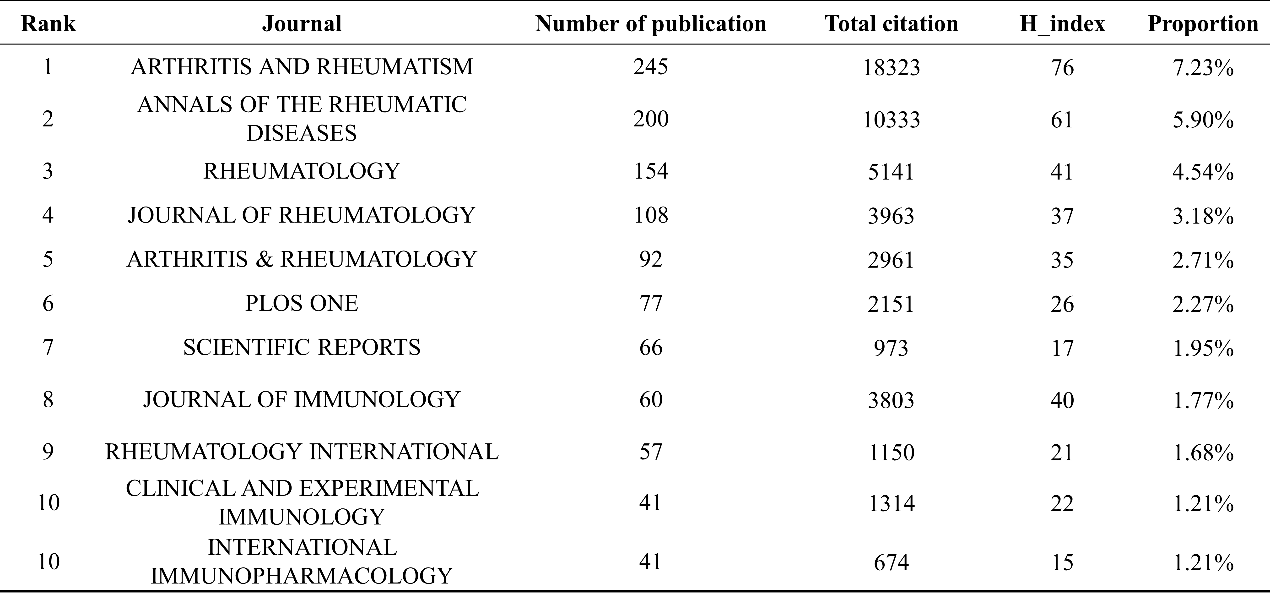


**Table S2.** Top 10 journals ranked by number of publications.


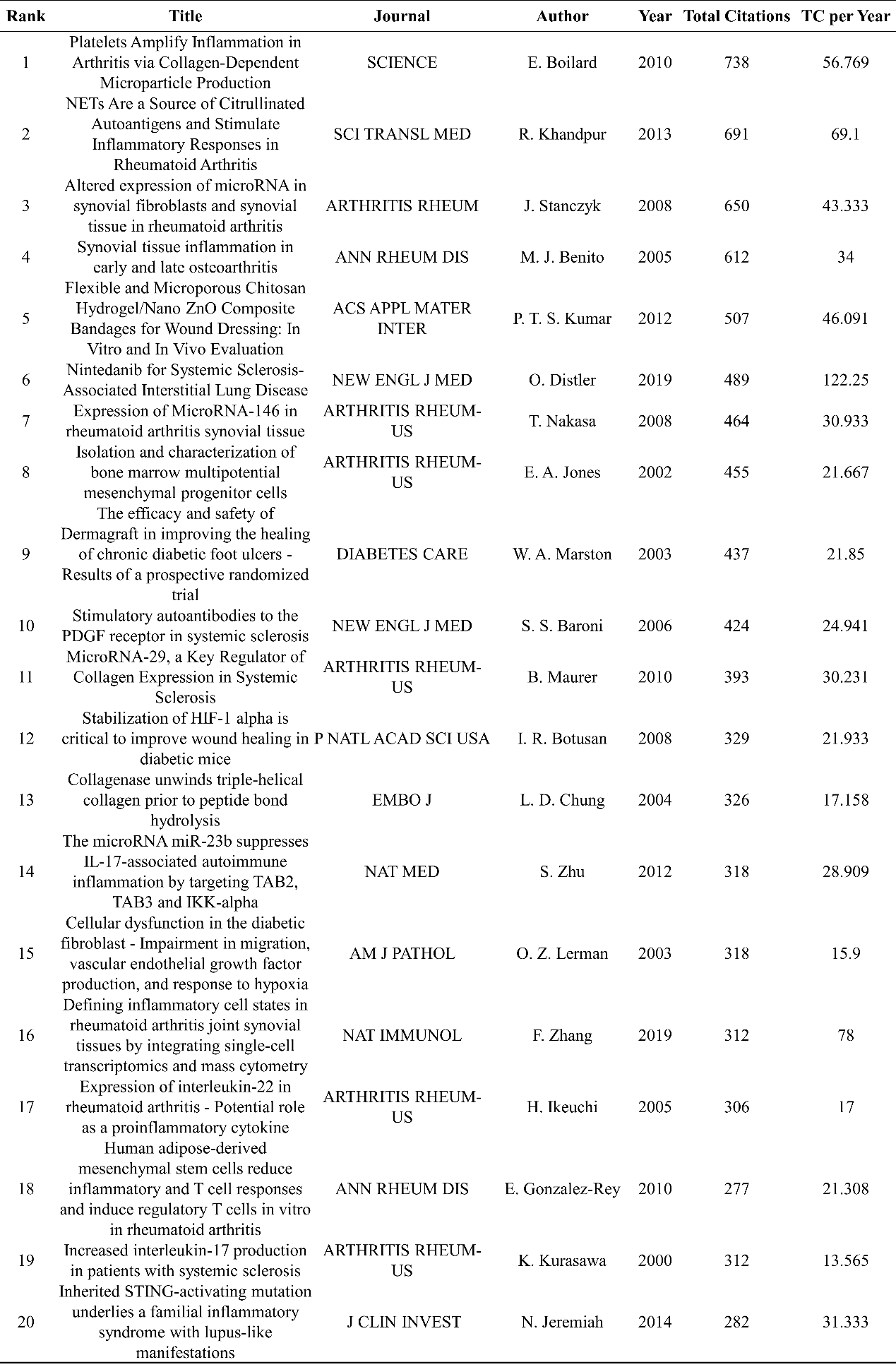


**Table S3.** Top 20 articles ranked by total citation in the 21st century.


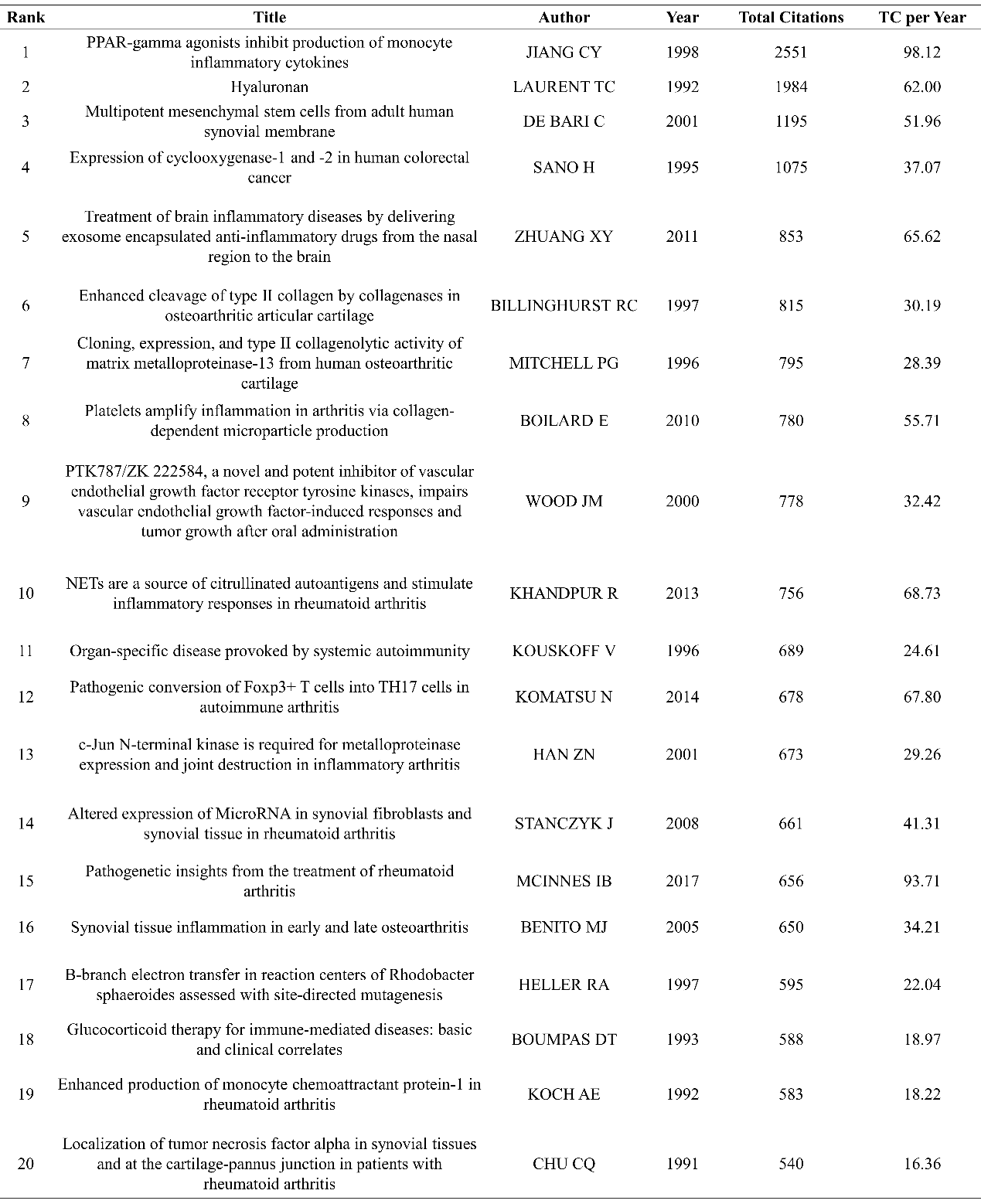


**Table S4.** Top 20 articles ranked by total citation in subgroup 1.


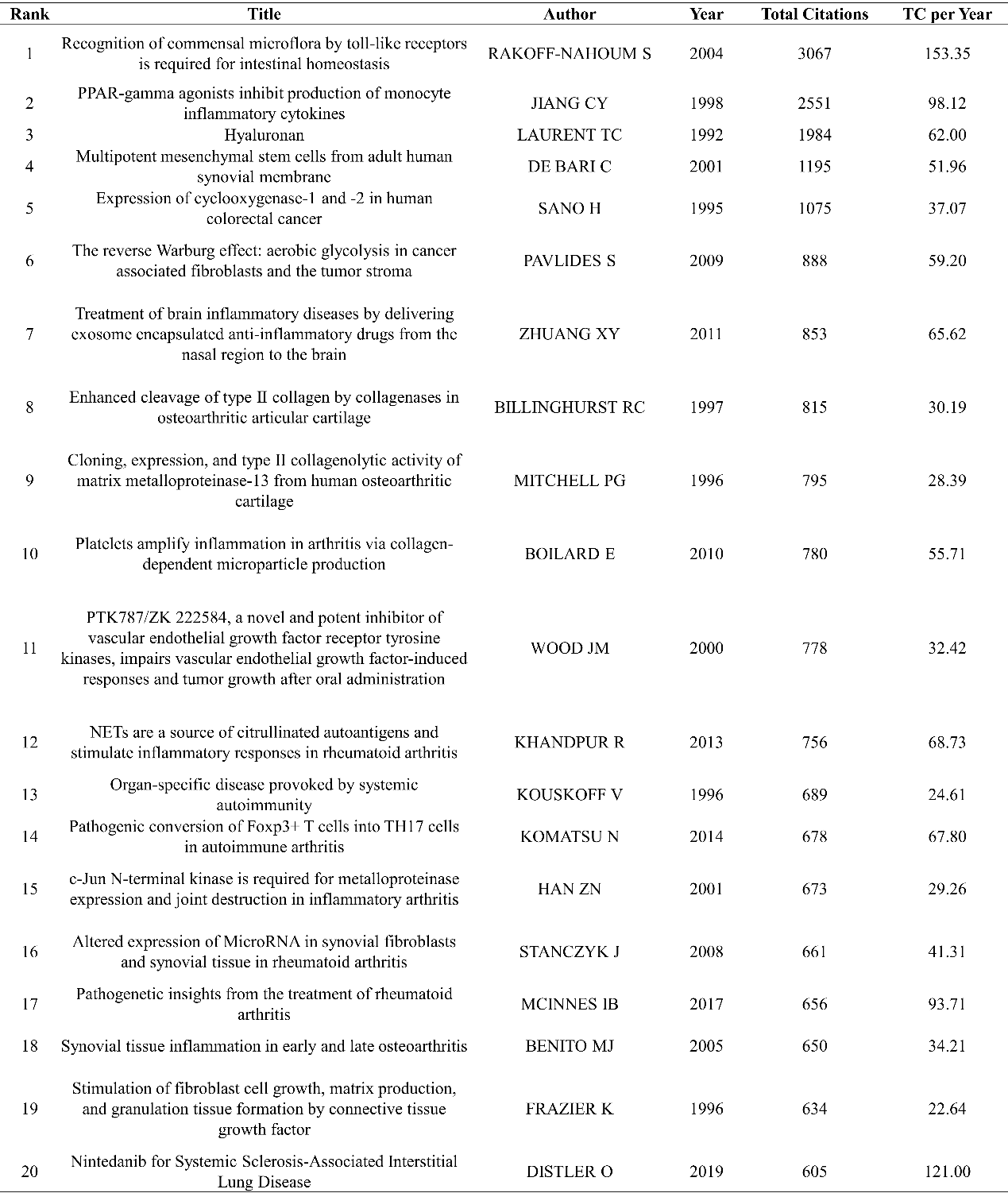


**Table S5.** Top 20 articles ranked by total citation in subgroup 2.
